# Long-term safety of brazikumab in the open-label period of a randomized phase 2a study of patients with Crohn’s disease

**DOI:** 10.1186/s12876-023-03078-7

**Published:** 2023-12-20

**Authors:** Silvio Danese, Andrew Beaton, Elizabeth A. Duncan, Anne-Kristina Mercier, Jessica Neisen, Henrik Seth, Sofia Zetterstrand, Bruce E. Sands

**Affiliations:** 1 Gastroenterology and Gastrointestinal Endoscopy Unit, Vita-Salute San Raffaele University, IRCCS San Raffaele Scientific Institute, Milan, Italy; 2 AstraZeneca, Cambridge, UK; 3 AstraZeneca, Durham, NC USA; 4 AstraZeneca, Gothenburg, Sweden; 5 Dr. Henry D. Janowitz Division of Gastroenterology, Icahn School of Medicine at Mount Sinai, New York, NY USA Box 1069, One Gustave L. Levy Place, New York

**Keywords:** Biologics, Crohn’s disease, Inflammatory bowel disease

## Abstract

**Background:**

Short-term efficacy and safety of brazikumab (MEDI2070), a human monoclonal antibody and anti-p19 subunit inhibitor of interleukin-23, was demonstrated in a phase 2a trial in patients with moderate-to-severe active Crohn’s disease (CD). We report brazikumab long-term safety and tolerability from the open-label period of this phase 2a study.

**Methods:**

Patients who completed the 12-week, double-blind induction period were eligible for inclusion in an open-label period where all patients received subcutaneous brazikumab (210 mg) every 4 weeks for 100 weeks. Patients had moderate-to-severe active CD and had failed or were intolerant to ≥ 1 anti-tumour necrosis factor alpha (TNFα) agent. Safety assessments included treatment-emergent adverse events (TEAEs); further assessments were pharmacokinetics and immunogenicity.

**Results:**

Of the 104 patients who entered the open-label period, 57 (54.8%) continued to the end of the open-label period and 47 (45.2%) discontinued brazikumab. The most common reasons for discontinuation were lack of response (14.4%), patient decision (12.5%), and TEAEs (11.5%). In total, 44 (84.6%) in the group switching from placebo to brazikumab (placebo/brazikumab) and 43 (82.7%) in the group continuing brazikumab (brazikumab/brazikumab) experienced 1 or more TEAEs. Most TEAEs were mild-to-moderate in severity. Common TEAEs included nasopharyngitis and headache. Numbers of treatment-emergent serious adverse events (TESAEs) were similar between groups. Infections occurred in 40.4% of patients in the placebo/brazikumab group and 50% in the brazikumab/brazikumab group. There were 5 TESAEs of infection, none of which were opportunistic. No major adverse cardiac events, malignancies, or deaths were reported.

**Conclusions:**

Brazikumab was well tolerated with an acceptable safety profile over a 100-week period in patients with moderate-to-severe active CD who failed or were intolerant to 1 or more anti-TNFα agents.

**Trial registration:**

NCT01714726; registered October 26, 2012.

## Introduction

The global prevalence of inflammatory bowel disease (IBD) is rising, affecting over 6 million people [1]. Crohn’s disease (CD) is one of the most prevalent forms of chronic IBD with symptoms including abdominal pain and diarrhea, which is sometimes accompanied by passage of blood and/or mucus [2]. Chronic bowel inflammation can progress to bowel obstruction due to stricture and fistula formation [3]. It can also be associated with extra-intestinal manifestations such as fatigue, anemia, arthropathy, osteoporosis, pyoderma gangrenosum, and erythema nodosum [2]. Patients with CD experience impaired quality of life, with their disease impacting both their personal lives and their work [4].

Commonly used medical therapies include aminosalicylates (eg, sulfasalazine and mesalamine), systemic corticosteroids, immunosuppressive agents (eg, azathioprine and methotrexate), antibacterial agents, and biologic agents (eg, adalimumab, infliximab, certolizumab, vedolizumab, and ustekinumab) [5–7]. Around one-third of patients with CD do not adequately respond to tumor necrosis factor alpha (TNFα) antagonists, and of those who do respond, approximately 50% lose response to therapy by 2 years [8]. Furthermore, disease heterogeneity presents a major challenge for treatment of CD [9].

Interleukin (IL)-23 is a proinflammatory cytokine involved in the maintenance of T helper type 17 (Th17) cells, which can contribute to the pathogenesis of CD [10]. IL-23 inhibition is an emerging strategy for the treatment of IBD, including CD. IL-23 consists of 2 subunits: p40 and p19 [11]. P40 is also a subunit of IL-12; therefore, p40 inhibitors such as ustekinumab, which is indicated for CD and UC [12], inhibit both IL-12 and IL-23 [13, 14]. Several antibodies have been designed to bind to p19 to specifically target IL-23, including mirikizumab, risankizumab, and guselkumab [15–17]. Risankizumab was recently approved in the United States as the first anti–IL-23/p19 antibody for the treatment of moderate-to-severe active CD [18].

Brazikumab is a human immunoglobulin G2 monoclonal antibody that selectively binds the p19 subunit of IL-23 [19]. In a phase 2a study in patients with moderate-to-severe CD who experienced treatment failure or were intolerant to ≥ 1 anti-TNFα agent, clinical improvement as measured by clinical response (decrease in Crohn’s Disease Activity Index [CDAI]) was observed after 8 weeks of brazikumab treatment [20]. Brazikumab was well tolerated during short-term treatment up to 24 weeks in the phase 2a study [20]. The objective of this analysis is to report the long-term safety and tolerability of brazikumab in patients with moderate-to-severe CD during the 100-week, open-label period of this phase 2a study (NCT01714726).

## Methods

### Study design

**Fig. 1:**
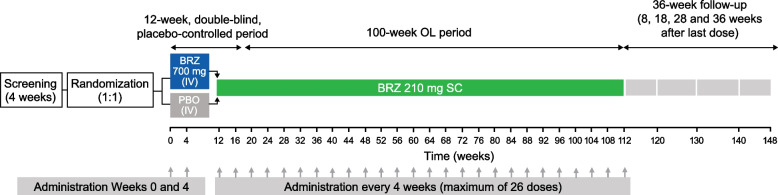
Study design. BRZ, brazikumab; IV, intravenous; OL, open-label; PBO, placebo; SC, subcutaneous

This phase 2a study consisted of a 12-week, double-blind, placebo-controlled treatment period in which patients received intravenous (IV) brazikumab 700 mg or placebo on days 1 and 29, a 100-week, open-label treatment period in which all patients received subcutaneous (SC) brazikumab 210 mg every 4 weeks (maximum of 26 dose administrations), and a 36-week, post-treatment follow-up period (Fig. 1). The overall methodology for the study and the results for the 12-week double-blind treatment period have been published previously [20]. This report describes the 100-week open-label period. The study was approved by the institutional review board/ethics committee before commencement and was conducted in compliance with the Declaration of Helsinki, the International Council on Harmonisation Guidance for Good Clinical Practice, and applicable regulatory requirements.

### Patients

Detailed inclusion and exclusion criteria have previously been published [20]. Briefly, patients who were 18–65 years old with a diagnosis of CD for at least 6 months prior to screening and a classification of moderate-to-severe active CD (defined as CDAI ≥ 220 and ≤ 450 at day 1) were included. Patients were required to have experienced treatment failure or been intolerant to anti-TNFα therapy, as determined by the investigator. The following concomitant medications were permitted: 5-aminosalicylates; prednisone up to 20 mg/day or equivalent; budesonide up to 6 mg/day; azathioprine; 6-mercaptopurine; methotrexate; oral antibiotics for CD (except for the treatment of acute illness); probiotics (eg, Culturelle, *Saccharomyces boulardii*), provided that the dose had been stable for the 2 weeks prior to baseline; and antidiarrheals (eg, loperamide, diphenoxylate with atropine) for control of chronic diarrhea. All patients provided written informed consent.

### Assessments

Safety and tolerability endpoints included treatment-emergent adverse events (TEAEs) and treatment-emergent serious adverse events (TESAEs), assessed every 4 weeks in the open-label period. TEAEs of special interest included, but were not limited to, infusion reactions, hypersensitivity reactions (eg, anaphylaxis), major adverse cardiac events (MACE; defined as myocardial infarction, stroke, or cardiovascular death), infections, and malignancies. Adverse events were evaluated at each study visit. Serum concentrations of brazikumab were determined at nine time points: both pre-dose and at the time of infusion at weeks 0 and 4, at week 8 (visit without dosing), pre-dose at weeks 12, 24 and 112, and 28 weeks after the last dose administration (follow-up visit). The presence of antidrug antibodies was assessed at weeks 0, 8, 24, and 112, and 28 weeks after the last dose administration (follow-up visit).

Long-term exploratory efficacy endpoints included clinical response (CDAI total score < 150 or reduction from baseline in CDAI score of ≥ 100 points) and clinical remission (CDAI total score < 150) assessed at weeks 56 and 112.

### Statistical analysis

Descriptive statistics for demographics and safety data were reported.

Nonresponder imputation was applied to dichotomous efficacy measures for missing data. The imputed nonresponders before week 8 were considered nonresponders for all subsequent visits. Per protocol, patients with a clinically meaningful increase in corticosteroid dose were also considered nonresponders. A clinically meaningful increase was defined as an increase of at least 5 mg/day for at least 3 days of prednisone, or equivalent, or an increase of at least 3 mg/day for at least 3 days of budesonide.

## Results

### Patient disposition

**Fig. 2:**
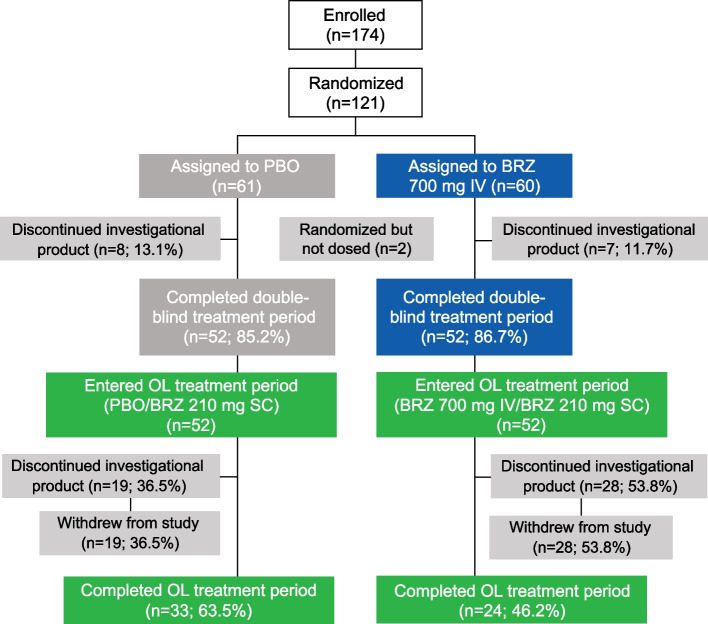
Patient disposition. BRZ, brazikumab; IV, intravenous; OL, open-label; PBO, placebo; SC, subcutaneous

A total of 104 patients received SC brazikumab 210 mg during the open-label period, of whom 52 previously received placebo (placebo/brazikumab group) and 52 previously received brazikumab (brazikumab/brazikumab group) during the 12-week double-blind phase (Fig. 2). Mean duration of exposure during the open-label period was 721 days in the placebo/brazikumab group and 630 days in the brazikumab/brazikumab group. A total of 47 (45.2%) patients discontinued brazikumab, with the most common reasons for discontinuation being lack of response (14.4%), patient decision (12.5%), and adverse events (11.5%). A total of 57 patients (54.8%) continued to the end of the open-label treatment period.

### Baseline demographics and clinical characteristics

Baseline demographics and clinical characteristics for patients entering the open-label period were similar for the group of patients previously randomized to brazikumab and those previously randomized to placebo (Table 1). Mean age of the population was 37 years, the majority (60.6%) of patients were women, mean disease duration was 12.6 years, and mean CDAI was 315.1. Most patients had previously used fewer than 3 anti-TNFα agents, approximately half were using corticosteroids, and 26.9% were using an immunomodulator at study baseline.

**Table 1:** Patient demographics and clinical characteristics at study baseline for patients entering the open-label period

	**Brazikumab/ Brazikumab (*n* = 52)**	**Placebo/ Brazikumab (*n* = 52)**	**Total (*N* = 104)**
Age, mean ± SD, y	35.3 ± 11.1	37.8 ± 10.6	36.5 ± 10.9
Female, n (%)	32 (61.5)	31 (59.6)	63 (60.6)
Weight, mean ± SD, kg	70.1 ± 21.0	71.9 ± 15.3	71.0 ± 18.3
Race, n (%)			
White	50 (96.2)	49 (94.2)	99 (95.2)
Non-White	2 (3.8)	3 (5.8)	5 (4.8)
Not Hispanic or Latino, n (%)	48 (92.3)	47 (90.4)	95 (91.3)
Disease duration, mean ± SD, y	13.0 ± 9.7	12.1 ± 8.7	12.6 ± 9.2
CDAI, mean ± SD, points^a^	321.9 ± 62.2	308.3 ± 55.8	315.1 ± 59.2
Prior use of anti-TNFα agents, n (%)			
1	18 (34.6)	16 (30.8)	34 (32.7)
2	32 (61.5)	31 (59.6)	63 (60.6)
≥ 3	2 (3.8)	5 (9.6)	7 (6.7)
Corticosteroid use at baseline, n (%)	28 (53.8)	26 (50.0)	54 (51.9)
Immunomodulator use at baseline, n (%)	15 (28.8)	13 (25.0)	28 (26.9)

^a^CDAI score ranges from 0 to 600, with higher scores indicating worse disease

CDAI, Crohn’s Disease Activity Index; SD, standard deviation; TNFα, tumor necrosis factor alpha

### TEAEs in the open-label period

The safety profile over the 100-week open-label period was consistent with the safety profile from the 12-week double-blind period (previously published [20]). Throughout the open-label period, 44 (84.6%) patients in the placebo/brazikumab group and 43 (82.7%) in the brazikumab/brazikumab group experienced at least 1 TEAE (Table 2.). The majority of TEAEs were mild to moderate in severity. TEAEs of grade 3 severity or higher were reported by 4 (7.7%) patients in the placebo/brazikumab group and 11 (21.2%) in the brazikumab/brazikumab group. No life-threatening or fatal TEAEs were reported. In all, 15.4% in the placebo/brazikumab group and 23.1% of patients in the brazikumab/brazikumab group reported 1 or more TESAEs. The total numbers of TESAEs were similar between treatment groups (12 in the placebo/brazikumab group and 16 in the brazikumab/brazikumab group). The most frequent TEAE for patients who switched from placebo to brazikumab was headache, followed by abdominal pain. The most frequent TEAEs for all patients who received brazikumab in the open-label period were nasopharyngitis, headache, and CD. TEAEs leading to discontinuation of brazikumab occurred in 12/104 (11.5%) patients in the total population. Injection site reactions occurred in 2 patients in the brazikumab/brazikumab group and did not lead to discontinuation.

**Table 2:** Summary of adverse events in the open-label period

Patients, n (%)	Brazikumab/ Brazikumab (*n* = 52)	Placebo/ Brazikumab (*n* = 52)	Total (*N* = 104)
≥ 1 TEAE	43 (82.7)	44 (84.6)	87 (83.7)
≥ 1 TESAE	12 (23.1)	8 (15.4)	20 (19.2)
TEAEs of ≥ grade 3 severity	11 (21.2)	4 (7.7)	15 (14.4)
TEAEs leading to study drug discontinuation	7 (13.5)	5 (9.6)	12 (11.5)
TEAEs leading to withdrawal from the study	0	1 (1.9)	1 (1.0)
Total number of TEAEs	387	444	831
TEAEs occurring in ≥ 10% of patients, n (%)^a^			
Headache	11 (21.2)	12 (23.1)	23 (22.1)
Nasopharyngitis	15 (28.8)	8 (15.4)	23 (22.1)
Abdominal pain	10 (19.2)	9 (17.3)	19 (18.3)
CD	11 (21.2)	6 (11.5)	17 (16.3)
Diarrhea	7 (13.5)	7 (13.5)	14 (13.5)
Influenza	5 (9.6)	8 (15.4)	13 (12.5)
Nausea	6 (11.5)	5 (9.6)	11 (10.6)
Vomiting	3 (5.8)	8 (15.4)	11 (10.6)
Pyrexia	3 (5.8)	6 (11.5)	9 (8.7)
Upper respiratory tract infection	1 (1.9)	8 (15.4)	9 (8.7)
Total number of TESAEs	16	12	28
TESAEs associated with GI disorders, n (%)			
CD	6 (11.5)	1 (1.9)	7 (6.7)
Abdominal pain	0	1 (1.9)	1 (1.0)
Anal fistula	1 (1.9)	0	1 (1.0)
Diarrhea	0	1 (1.9)	1 (1.0)

^a^By preferred term, MedDRA version 19.1. CD, Crohn’s disease; GI, gastrointestinal; TEAE, treatment-emergent adverse event; TESAE, treatment-emergent serious adverse event

### TEAEs of special interest

No infusion reactions occurred during the open-label period. Two hypersensitivity reactions occurred, both in the brazikumab/brazikumab group. No MACE occurred during the study. Throughout the open-label period, 40.4% of patients in the placebo/brazikumab group and 50% in the brazikumab/brazikumab group experienced infections. The most frequently reported infections were urinary tract infection, vulvovaginal mycotic infection, sinusitis, bronchitis, and upper respiratory tract infection (Table 3). There were 5 cases of TESAEs of infection, 1 of which led to treatment discontinuation, and there were no TESAEs of opportunistic infections. No malignancies were reported during the study.

**Table 3:** Infections in the open-label period

AE, count	Brazikumab/ Brazikumab (*n* = 52)	Placebo/ Brazikumab (*n* = 52)	Total (*N* = 104)
Total infections^a^	42	58	100
Urinary tract infection	4	5	9
Vulvovaginal mycotic infection	1	6	7
Sinusitis	1	6	7
Bronchitis	2	4	6
Upper respiratory tract infection	2	4	6
Tonsilitis	2	3	5
Tooth infection	3	2	5
Subcutaneous abscess	4	0	4
*Clostridium difficile* colitis	3	0	3
Pharyngitis streptococcal	1	2	3
Anal abscess	2	0	2
Anal candidiasis	0	2	2
Ear infection	0	2	2
Groin abscess	1	1	2
Influenza	1	1	2
Nasopharyngitis	1	1	2
Pharyngitis	2	0	2
Pyelonephritis	1	1	2
Tooth abscess	0	2	2

Infections occurring in 1 patient each for the brazikumab/brazikumab group included abdominal abscess, *Campylobacter* infection, cholecystitis, external ear inflammation, gingivitis, mastoiditis, esophageal candidiasis, pilonidal cyst, postprocedural infection, rhinitis, and viral infection

Infections occurring in 1 patient each for the placebo/brazikumab group included amoebiasis, *Cryptosporidiosis* infection, diarrhea, folliculitis, gastroenteritis, herpes zoster, infected dermal cyst, pelvic abscess, peritonitis, pneumonia, pyrexia, respiratory tract infection, respiratory tract infection (viral), sialadenitis, tracheobronchitis, and vomiting

^a^Infections were defined as any events involving a suspected viral, bacterial, fungal, or other infectious agent, including viral reactivation events and opportunistic infections, meeting ≥ 1 of the following criteria: events that are serious, grade 3 or higher, involve treatment with oral or parenteral antibiotics/antivirals/antifungals, or involve study discontinuation

AE, adverse event

### Pharmacokinetics and immunogenicity

**Fig. 3:**
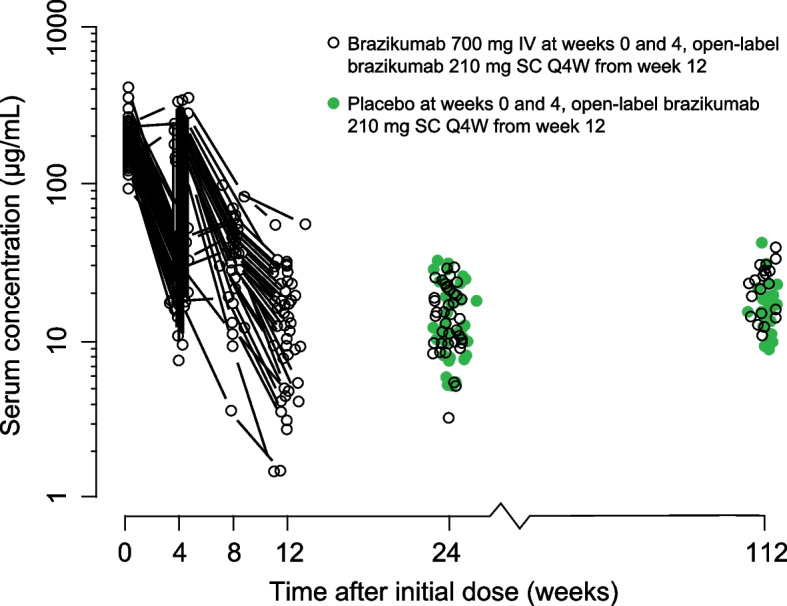
Individual serum concentration–time profiles of brazikumab. IV, intravenous; Q4W, every 4 weeks; SC, subcutaneous

The maximal mean serum concentration of brazikumab (209 μg/mL) was observed after the end of the second infusion of 700 mg at week 4. Trough levels during the SC administration open-label period (determined at 24 and 112 weeks) were generally comparable (mean values ranging from 14.5 to 22.4 μg/mL) and similar to the mean pre-dose concentration at week 12 (16.7 μg/mL) at the end of the IV induction period. Trough serum concentrations at week 24 and 112 in patients who had initially received 700 mg IV doses during the induction period were similar to those who initially received placebo (15.1 μg/mL vs 14.5 μg/mL, and 22.4 μg/mL vs 18.3 μg/mL, respectively) (Fig. 3). Post-baseline antidrug antibodies were detected in 2 patients, but were transient and non-neutralizing.

### Efficacy

**Fig. 4:**
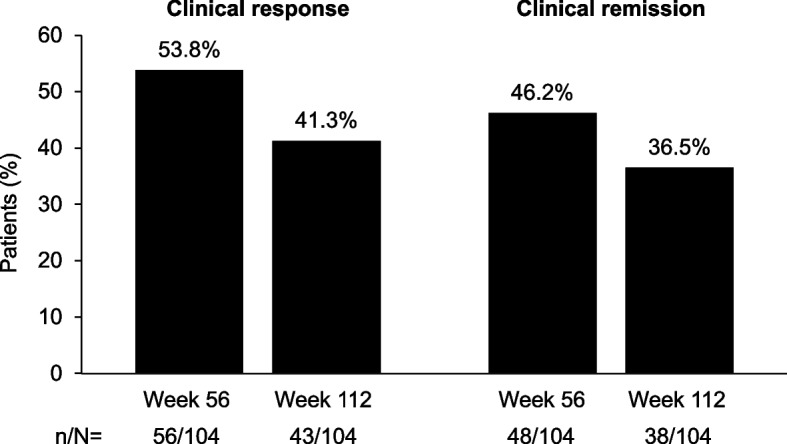
Clinical response and remission rates in patients receiving brazikumab during the open-label period. Nonresponder imputation used for missing data. Clinical response was defined as a CDAI total score < 150 or reduction from baseline in CDAI score of ≥ 100 points. Clinical remission was defined as a CDAI total score < 150. CDAI, Crohn’s Disease Activity Index

Clinical response was observed in 53.8% (56/104) and 41.3% (43/104) of patients at weeks 56 and 112, respectively (Fig. 4). Clinical remission rates for weeks 56 and 112 were 46.2% (48/104) and 36.5% (38/104), respectively (Fig. 4).

## Discussion

We have previously shown results from this study in which brazikumab demonstrated efficacy versus placebo; a CDAI response (defined by either a CDAI score of  < 150 or a CDAI reduction from baseline of ≥ 100 points) was achieved by 49.2% of patients receiving brazikumab versus 26.7% of patients receiving placebo at week 8. At week 24, 53.8% of patients in the brazikumab/brazikumab group and 57.7% of patients in the placebo/brazikumab group achieved CDAI response [20]. Here, we show that brazikumab 210 mg SC was well tolerated and showed an acceptable safety profile over 100 weeks in patients with CD refractory to TNFα antagonists, with no new safety signals. The most common TEAEs in both patients who switched from placebo to brazikumab and patients who continued brazikumab were headache, nasopharyngitis, and abdominal pain. Approximately half of the TESAEs were gastrointestinal related, the majority of which were related to CD. No TESAEs of opportunistic infections were observed. Immunogenicity of brazikumab was low; antidrug antibodies were only detected in 2 patients and were transient and non-neutralizing.

Recent phase 2/3 studies of other IL-23 inhibitors in patients with CD have shown similar safety profiles. Common TEAEs in a study of mirikizumab over a 52-week period included headache and nasopharyngitis [15]. Reported opportunistic infections and SAEs in the study were low and there were no deaths or malignancies. In an open-label extension study of risankizumab that followed 65 patients for up to 196 weeks, common TEAEs included nasopharyngitis, gastroenteritis, and fatigue, and SAEs occurred in 35.4% of patients [16]. Infections were reported in most patients, of which only 6 were serious. Opportunistic infections occurred in 3 (4.6%) patients. Hepatic events occurred in 6 (9.2%) patients and included elevated liver enzymes and a single case of hepatic steatosis. All were assessed as grade 1. No deaths, malignancies, or MACE were reported. Rates of serious infections reported in phase 3 trials were low and comparable between placebo and risankizumab groups [21] or slightly lower in risankizumab groups than in placebo-treated patients [22]. Opportunistic infections occurred in 1% or less of patients in all groups [21, 22]. Data from a 12-week, placebo-controlled study of guselkumab showed similar proportions of TEAEs between placebo- and guselkumab-treated patients [17]. As in the other studies, headache and nasopharyngitis were the most common TEAEs. SAEs occurred in 8 (3.7%) guselkumab-treated patients. Infections occurred in 33 (15.1%) guselkumab-treated patients over the 12-week period and serious infections occurred in 3 (1.4%) patients. Data from trials of IL-23 inhibitors in psoriasis and psoriatic arthritis have also shown low rates of serious infections [23, 24]. Overall, these studies suggest a favorable safety profile of IL-23 inhibitors. There also does not appear to be an increased risk of serious infections or malignancies with IL-12/IL-23 inhibition; phase 3 trials and real-world studies of ustekinumab have found low rates of serious infections and malignancies [14, 25–27].

In addition to the favorable safety profile, longevity of clinical response and remission rates were observed out to week 112 (41.3% and 36.5%, respectively). Although encouraging of a lasting therapeutic benefit to participants, these data should be viewed cautiously given the open-label nature of the extended treatment period and the lack of a placebo control arm for comparison.

The current study is one of the longest studies to evaluate the safety of an IL-23 inhibitor in patients with CD. Taken together with long-term risankizumab safety data, these results indicate a consistent safety profile over long-term use for IL-23 inhibitors. Future studies on the safety of brazikumab for a longer treatment duration are warranted. Additionally, further analyses of long-term efficacy of brazikumab are needed.

### Limitations

The small sample size and patient population limited to those with inadequate biologic response limit generalizability of the results. The open-label design and lack of placebo comparator are also a limitation of this study. Additionally, endoscopy was not performed.

## Conclusions

In this 100-week, open-label period, brazikumab was well tolerated in patients with moderate-to-severe active CD who experienced treatment failure or were intolerant to ≥ 1 anti-TNFα agent, warranting future studies in broader patient populations.

## Data Availability

Data underlying the findings described in this manuscript may be obtained in accordance with AstraZeneca’s data sharing policy described at https://astrazenecagrouptrials.pharmacm.com/ST/Submission/Disclosure. Data for studies directly listed on Vivli can be requested through Vivli at www.vivli.org. Data for studies not listed on Vivli could be requested through Vivli at https://vivli.org/members/enquiries-about-studies-not-listed-on-the-vivli-platform/. AstraZeneca Vivli member page is also available outlining further details: https://vivli.org/ourmember/astrazeneca/.

## Abbreviations

CD: Crohn’s disease
CDAI: Crohn’s Disease Activity Index
IL: Interleukin
IV: Intravenous
MACE: Major adverse cardiac events
SAEs: Serious adverse events
SC: Subcutaneous
TEAEs: Treatment-emergent adverse events
IBD: Inflammatory bowel disease
Th17: T helper type 17
TNFα: Tumor necrosis factor alpha
